# Maternal responses to infant positive affect and toddler regulation of positive affect: A longitudinal pilot study

**DOI:** 10.1002/imhj.70124

**Published:** 2026-11-13

**Authors:** Gabrielle Schmitt, Brittany Jamieson, Leslie Atkinson

**Affiliations:** ^1^ Department of Psychology Toronto Metropolitan University Toronto Canada; ^2^ Department of Psychiatry and Behavioural Neurosciences McMaster University Hamilton Canada

**Keywords:** delay of gratification, emotion regulation, emotion socialization, infancy, positive affect, toddlerhood

## Abstract

Children's ability to regulate positive affect (PA) is a crucial yet understudied aspect of early socioemotional development. This pilot study investigated whether maternal responses to infant PA longitudinally predict toddlers’ regulation of PA. Participants were 32 mother–infant dyads from a larger longitudinal study in Toronto, Canada. At 11–12 months, dyads were video recorded during naturalistic play at home. Maternal responses to infant PA were assessed using an observational coding system that distinguishes supportive (e.g., elaborative, matching) from nonsupportive (e.g., intrusive, ignoring) responses. At 18–20 months, toddlers completed the gift delay task, which measures regulation of high‐arousal PA through latency to touch a desirable toy. Multiple regression analysis controlling for age and family income showed that a greater proportion of supportive responses in infancy predicted better regulation of PA in toddlerhood (β = .37, *p* = .027). Findings provide preliminary evidence that supportive maternal responses to PA in infancy are associated with capacities to regulate PA in toddlerhood. Results highlight the importance of examining positive as well as negative affect in models of early emotion regulation and suggest potential targets for early intervention.

## 1 INTRODUCTION

Learning how to manage emotions is essential for children's mental health (Beauchaine & Cicchetti, 2019; Cicchetti et al., 1995). Children who struggle to regulate their emotions are at increased risk for emerging psychopathology, including internalizing (e.g., depression, anxiety) and externalizing (e.g., rule‐breaking, aggression) problems (Aldao et al., 2016; Cavicchioli et al., 2023). Accordingly, researchers have devoted significant attention to understanding how children develop emotion regulation abilities. From a clinical perspective, identifying environmental factors that support or impede this development is particularly important, as such factors provide modifiable targets for prevention and early intervention.

**Key Findings**

Maternal responses to infant positive affect may contribute to toddlers’ emotion regulation abilities, an important aspect of socioemotional development.

Infants who received more supportive maternal responses showed better regulation of positive affect in toddlerhood, as measured in a delayed gratification task.

**Statement of Relevance**

For infant and early childhood mental health practitioners, results highlight the importance of supporting positive emotions within the caregiver–child relationship. Helping caregivers notice, reciprocate, and amplify infants’ positive emotions may promote young children's socioemotional health.

Although there is much debate about how to define *emotion regulation*, most researchers agree that it involves altering or maintaining an emotion (as internally experienced or outwardly expressed) to achieve situation‐specific goals (Cole et al., 1994; Diamond & Aspinwall, 2003; Gross, 2015; Thompson, 1994; Yap et al., 2008). Historically, research has focused on the regulation of negative affect—how children learn to manage sadness, anger, and fear. Relatively less is known about environmental factors that support children's regulation of positive affect (PA), such as joy and excitement (Doan et al., 2023). This gap is notable because evidence suggests that the regulation of PA is a distinct yet equally important facet of early self‐regulation (Davis & Suveg, 2014; Shin et al., 2023). Effective regulation of PA enables children to sustain or enhance positive emotion when desirable (e.g., maintaining excitement while anticipating a reward), but also inhibit or delay its expression when necessary to meet task demands or social expectations (e.g., waiting to open a gift; Davis & Suveg, 2014).

### 1.1 Parental socialization of PA

Parents play a foundational role in children's development of emotion regulation (Morris et al., 2007). *Emotion socialization* encompasses parenting behaviors that influence a child's learning about the expression and regulation of emotions (Eisenberg, 2020; Eisenberg et al., 1998). One key mechanism of influence is how parents respond to children's emotional displays. These responses are often conceptualized as *supportive* or *nonsupportive* in existing theory and research (Doan et al., 2023). Supportive responses to PA—such as reciprocating a child's smile, celebrating an achievement, or elaborating on a pleasurable experience—communicate that positive emotions are valued and encourage children to express and savor them. Frequent supportive responses may reinforce the use of PA for adaptive coping and goal‐directed behavior (Diamond & Aspinwall, 2003; Doan et al., 2023). In contrast, nonsupportive responses—such as ignoring, minimizing, or interrupting displays of PA—communicate that positive emotions are less worthy of attention and discourage their expression, which limits opportunities to practice effective PA regulation (Fredrick et al., 2019; Yap et al., 2008). Over time, these response patterns shape how children express and manage PA within the parent–child relationship and provide a template for the regulation PA in a variety of settings (Kiel & Kalomiris, 2015).

Infancy may be a particularly sensitive period for the socialization of PA. During the first year of life, infants have limited capacity to regulate emotions independently and rely heavily on caregivers to scaffold their emotional experience (Feldman, 2003; Taraban & Shaw, 2018). Infants gradually internalize these abilities, with individual differences in emotion regulation becoming increasingly apparent by toddlerhood and continuing to differentiate across childhood (Diamond & Aspinwall, 2003). Because early interactions with caregivers lay the foundation for emerging self‐regulation, parental responses to PA may be especially important during this period. Yet, to our knowledge, no studies have systematically examined whether parental responses to infant PA are associated with children's later ability to regulate PA.

### 1.2 Empirical evidence linking parental responses to PA with child regulation of PA

Only a few studies have directly tested whether parental responses to PA predict children's emotion regulation. Most of this research involves school‐age children or adolescents and has produced inconclusive findings. In a cross‐sectional sample of preadolescents (11–13 years), Yap et al. (2008) assessed maternal responses to PA using both self‐report and observational methods. The observational coding system focused on two types of nonsupportive responses (i.e., punishing and dysphoric responses to child PA) within the context of a positive event planning task. Mothers’ self‐report of nonsupportive responses and observed dysphoric responses were associated with children's maladaptive regulation of negative affect. Notably, however, Yap et al. (2008) did not examine children's regulation of PA as an outcome.

In a slightly younger sample (7–12 years), Moran et al. (2019) assessed maternal responses to child PA using self‐report measures and transcript‐coded interactions. Mother–child dyads engaged in a 5‐min discussion about a recent positive event, which was transcribed and coded for maternal responses to children's PA words. The coding system identified four types of verbal responses: validation, elaboration, questioning, and ignoring. Neither mother‐reported nor transcript‐coded responses were significantly associated with children's regulation of PA. One possible explanation for these null findings is the coding approach, which focused exclusively on linguistic content and therefore did not capture nonverbal cues (e.g., facial expressions, vocal tone, timing) that may be important indicators of supportive responding.

Fredrick et al. (2019) observationally coded mothers’ supportive responses to adolescents’ PA during a 5‐min planning task in which dyads were asked to plan a fun family trip. Supportive responses included enthusiasm (i.e., expressing interest, excitement, or joy) and elaboration (i.e., seeking information, asking questions, or providing details). As hypothesized, supportive responses concurrently predicted adolescents’ effective regulation of PA. To our knowledge, this is one of the only published studies to detect a significant association between observed maternal responses and children's PA regulation. The use of a composite score that combined diverse forms of supportive responding, rather than focusing on individual response categories, may have contributed to this finding.

More recently, Shin et al. (2023) coded parental coaching of preschool children's PA during a task that involved discussing an upsetting event for 2.5 min. Parents were assigned a global coaching score based on their encouragement (e.g., labelling, validation, mirroring) of child PA during the task. Contrary to expectations, parental coaching of PA *negatively* predicted children's self‐regulation in a multiple regression analysis (although the corresponding correlation was small and nonsignificant, raising the possibility of a suppression effect). This unexpected finding may be related to the assessment of global self‐regulation as an outcome, rather than PA regulation specifically. It is also noteworthy that parental coaching of PA was observed during a negative emotion discussion, a context in which children's PA may occur infrequently and its encouragement may serve a different function.

### 1.3 Gaps in the literature

Taken together, the methodology of existing research limits our understanding of how parental responses to children's PA contribute to emotion regulation. First, most studies have relied on cross‐sectional designs with school age children, making it unclear whether parental responses to PA predict regulatory capacities earlier in development. Second, findings may lack ecological validity due to the use of highly structured laboratory tasks and brief observation windows (e.g., 5 min) to assess parental responses. Third, observational coding systems have often been narrow in scope, focusing on specific response types (e.g., punishing and dysphoric responses) or linguistic content alone, which may not provide a complete picture of supportive and nonsupportive responding. There remains a need for longitudinal research using ecologically valid observations and comprehensive coding systems that capture a broader range of behaviors relevant to PA socialization.

Related research in infancy has addressed some of these methodological limitations by using prospective designs and naturalistic observations. For example, longitudinal studies have shown that maternal matching of infant affect during face‐to‐face interactions predicts increases in infant PA over time (Malatesta et al., 1986; Nicely et al., 1999). More recently, Davis et al. (2019) found that maternal displays of PA during feeding and play predicted increases in infant PA across the first year of life. While these findings suggest that the socialization of PA begins in infancy, the literature has not examined diverse maternal responses to infant PA (beyond affective matching), nor has it assessed children's *regulation* of PA as a developmental outcome.

### 1.4 The current study

The present study addresses these gaps by evaluating the predictive validity of a novel observational coding system designed to capture a broad range of maternal responses to infant PA. The coding system was applied to naturalistic home‐based observations at 11–12 months, enhancing ecological validity. Children's ability to regulate PA was subsequently assessed at 18–20 months using the gift delay task (Kochanska et al., 1996), a paradigm that elicits high‐arousal PA and requires children to inhibit their approach toward a desirable object. Latency to touch the gift served as a behavioral indicator of PA regulation, reflecting the child's ability to delay immediate gratification and manage excitement. We hypothesize that infants who receive a greater proportion of supportive responses to PA exhibit better regulation of PA in toddlerhood.

## 2 METHOD

### 2.1 Participants

Participants were drawn from the Kids‐in‐Context Study (Gu et al., 2025; Schmitt et al., 2025), a prospective community sample of 144 mother–infant dyads in Toronto, Canada. Mothers were recruited through presentations at community centers and parenting events, social media platforms, and flyer postings across the city. Dyads were eligible for participation if (a) the mother was proficient in English and at least 18 years of age, (b) the dyad had no major medical conditions, and (c) the infant's birth weight was over 2500 g (as reported by mother).

The current study involves the subsample of 32 dyads who completed all assessments prior to the COVID‐19 pandemic and participated in the gift delay task, which was discontinued following the pandemic onset. Data were collected during home visits at three assessment periods: early infancy (*M* = 6.55 months, *SD *= .49), late infancy (*M* = 12.11 months, *SD* = .52), and toddlerhood (*M* = 19.42, *SD* = .58).

At the initial assessment, mothers ranged in age from 26 to 43 years (*M* = 34.24, *SD* = 4.12). There were 17 male and 15 female infants. Most mothers were university educated (88%) and either married or in a common law relationship (88%). Regarding race/ethnicity, the majority of mothers were White (*n* = 26), while a minority were biracial (*n* = 4) or Asian (*n *= 2). White mothers were overrepresented in the current sample (81%) relative to the broader Toronto population (49%; Statistics Canada, 2017). Annual family income before tax ranged from 10,000–15,000 to over 250,000 CAD, with a median income in the 100,000–150,000 CAD bracket. When data were collected, the median family income in Toronto was 110,800 CAD per year (Statistics Canada, 2024).

### 2.2 Procedure

Home visits were conducted between June 2018 and March 2020. All procedures were approved by the Research Ethics Board at Toronto Metropolitan University. Mothers provided written informed consent prior to participation. The current study focuses on observational data collected in late infancy (11–12 months) and toddlerhood (18–20 months).

#### 2.2.1 Mother–infant play session

At 11–12 months, mother–infant dyads were video recorded in their home during 30 min of play. Each dyad was provided with a standardized set of toys (i.e., musical phone, musical drum with blocks, toy car, sensory ball), and mothers were instructed to interact with their infant as they normally would. The interaction consisted of three 10‐min episodes. In the first episode, mothers completed questionnaires on an iPad while their infant played freely. In the second episode, mothers interacted with their infant using the provided toys, and in the final episode, they interacted without use of toys. These three episodes permitted observation of maternal behavior under various conditions (i.e., with and without the competing demands of another task; with and without mediating objects; Smith & Pederson, 1988) and were intended to mimic real‐life conditions. Dyads were permitted to move around their home during the play session since they could be followed with the camera. This procedure was designed to maximize ecological validity relative to static observations and split‐screen laboratory methods, but it also meant that at times, facial expressions were obscured. When both the mother and infant's faces could not be recorded in the same frame, the infant's face was prioritized.

#### 2.2.2 Toddler gift delay task

At 18–20 months, toddlers were video recorded in their home as they participated in a gift delay task. Each child was seated either in a highchair or on the floor. An experimenter showed the child a gift (i.e., animal bath toy), placed it under a transparent container, and stated: “This is a toy for you. I need to do something. I'll be right back! Do *not* touch the toy until I get back. Don't touch it. Do you understand? Don't touch it.” The experimenter then walked away and pretended to rummage through a backpack in the corner of the room, keeping their back toward the child. The task ended once 2 min elapsed or the child touched the toy (whichever came first). If 2 min elapsed, the experimenter returned to the child and said, “Okay, you can take the toy now.”

### 2.3 Observational coding

The play sessions were coded for maternal responses to infant PA in BORIS software (Friard & Gamba, 2016). This required a two‐step coding process: (1) identifying infant displays of PA and (2) classifying subsequent maternal responses. Given that infant initiations or changes in behavior are critical for eliciting maternal responses (Nicely et al., 1999; Van Egeren et al., 2001), PA was only coded if it represented a clear *change* in the infant's affective state (e.g., following a neutral state, the infant began to smile). Coders watched the videos in real time and marked the *onset* (i.e., start time) of infant PA. Once all PA was identified, coders returned to the marked onset times and coded a maternal response within a latency window of 3 s. Prior research investigating maternal response latencies suggests that most mothers respond to infant cues within 1–2 s, and nearly all responses occur within 3 s (Gros‐Louis et al., 2006; Nicely et al., 1999; Van Egeren et al., 2001).

#### 2.3.1 Infant PA

PA was operationalized as infant displays of high‐arousal positive emotion (e.g., joy, excitement, enthusiasm, surprise). During periods of play, low‐arousal positive emotion (e.g., calmness, contentment) typically reflects the infant's baseline affective state, and thus, is not likely to elicit a maternal response. Indicators of PA were selected based on existing measures of observed infant affect (Davis et al., 2019; Feldman, 2003; Izard, 1995; Olino et al., 2011). Specifically, PA was coded each time the infant displayed one or more of the following: a smile; a laugh or chuckle; a positive vocalization (e.g., squeal of excitement); an open mouth or gasp in surprise.

PA was identified by a team of four independent coders (i.e., one graduate student and three undergraduate research assistants). To establish initial reliability, 20% of videos were randomly selected and independently coded by all team members. The remaining videos were independently coded by two team members (assigned randomly) to minimize the possibility of overlooking any PA displays. Kappa coefficients for each coder pairing ranged from .75 to .89. Coding differences were resolved by consensus during weekly team meetings. The mean number of PA displays identified per video was 13.81 (*SD* = 5.93).

#### 2.3.2 Maternal responses to infant PA

Maternal responses to PA were classified using a coding system derived from prior measures (e.g., Lavelli & Fogel, 2013; Martins et al., 2018; Nicely et al., 1999), modified for infant age and the play context (Schmitt et al., 2025). Each response was broadly categorized as supportive or nonsupportive of the infant's PA display. The supportive response category included three codes: *emotion‐focused response* (emotional labeling, narration, or praise); *matching response* (reciprocal smiling or laughter); and *emotion amplification* (exaggerated expressions, playful teasing, or use of humor). The nonsupportive category included four codes: *intrusive response* (interrupting, redirecting, or raising concerns); *minimization* (low engagement or restricted affect); *ignoring* (no response); and *not attending* (unawareness of infant PA). If the mother could not see the infant's PA, or her response was not visible to coders, it was classified as *unobservable* and excluded from further analysis. The mean percentage of unobservable responses per video was 6.64% (median = 0%, interquartile range = 0%–13%).

Maternal responses were classified by two undergraduate research assistants, following extensive training on the coding system by the first author. Given that there were eight mutually exclusive codes, most of which occurred infrequently, Kappa coefficients were unstable when only 20% of videos were used to assess interrater reliability. Thus, all videos were double coded to ensure reliability estimates were stable. Kappa coefficients for each code ranged from .69–.89. Coding differences were reviewed weekly and resolved by consensus. To account for variation in the number of PA displays and thus maternal responses coded per dyad, proportion scores were computed for analysis. Specifically, the number of supportive responses was divided by the total number of observed responses (i.e., supportive + nonsupportive). Possible scores ranged from 0%–100%, with higher scores indicating a greater proportion of supportive responses (and a lower proportion of nonsupportive responses) to infant PA.

#### 2.3.3 Toddler regulation of PA

The gift delay task was coded for toddlers’ ability to regulate PA. Specifically, latency to touch the gift was recorded in seconds. If the child refrained from touching the gift for the full duration of the task, a latency score of 120 s was recorded. Possible scores ranged from 0–120 s, with higher scores representing better regulation of PA. Latency times were coded by one graduate student and two undergraduate research assistants. Given the small number of available videos, all videos were double coded to establish interrater reliability. Intraclass correlation coefficients indicated perfect reliability between coders (ICC = 1.0).

### 2.4 Sociodemographic covariates

Family income and toddler age were included as covariates in the primary analysis. Socioeconomic status has been associated with both maternal emotion socialization and child emotion regulation (Brophy‐Herb et al., 2013; Shaffer et al., 2012) and thus may confound the relationship between these two variables. In the present study, mothers reported their annual family income before tax on a 13‐point scale ranging from 1 (*less than $5000*) to 13 (*more than $250,000*). Toddler age was also selected as a covariate because emotion regulation, including delay of gratification, tends to improve as children age (Houck & Lecuyer‐Maus, 2004).

### 2.5 Data analytic plan

To address our hypothesis, a multiple regression analysis was conducted in R v. 4.4.0 (R Core Team, 2024). Toddler latency to touch the gift was the outcome variable, and the proportion of supportive maternal responses during infancy was the focal predictor variable. Annual family income and toddler age were included as covariates.

## 3 RESULTS

### 3.1 Descriptive information and preliminary analyses

Five mother–infant dyads were excluded from analyses because the play session was cut short or not administered (*n* = 2); the majority of maternal responses to infant PA were unobservable (*n* = 2); or the gift delay task ended early due to toddler fussiness (*n* = 1). This reduced the final sample to 27 dyads.

Means, standard deviations, and correlations among study variables are presented in Table 1. On average, mothers displayed a greater proportion of supportive (66%) than nonsupportive (34%) responses to infant PA. Maternal supportive responses did not significantly differ based on infant sex, *t*(25) = .09, *p* = .93, *d* = .03. There were also no sex differences in toddlers’ performance on the gift delay task, *t*(25) = .04, *p* = .97, *d* = .02. Approximately one third (33%) of toddlers touched the gift within the first 15 s, while one quarter (26%) refrained from touching the gift for the full 120 s. This resulted in a mild platykurtic distribution for toddler delay times (*z* = –1.77, *p* = .08). All other variables were normally distributed. Although not statistically significant, sociodemographic variables were moderately correlated with maternal supportive responses and/or toddler delay times in the expected directions (*r* = .23 to .29); the lack of statistical significance may be related to the small sample size.

**TABLE 1:** Means, standard deviations, and correlations across study variables.

Variable	*M*	*SD*	1	2	3	4
1. Supportive responses	66.40	22.54	—			
2. Toddler delay times	54.63	46.27	.38^*^	—		
3. Toddler age (months)	19.45	0.60	–.03	.23	—	
4. Annual family income			.29	.23	.30	—

*Note: N* = 27.

^*^ *p* < .05.

### 3.2 Statistical assumptions and outliers

Statistical assumptions for multiple regression were examined, including (a) normality of the model residual distribution, (b) linearity of partial relationships between each predictor and the model residuals, and (c) homoscedasticity of variance. The distribution of studentized residuals did not significantly deviate from normality according to the Shapiro‐Wilk test (*W* = .94, *p* = .10); however, visual inspection of the histogram and Q–Q plot suggested mild platykurtosis. No violations of linearity or homoscedasticity were observed. To address minor non‐normality in a small sample, robust standard errors (HC3) were used for hypothesis testing.

Multivariate outliers were assessed using Cook's distance, which combines leverage and residual size to quantify the influence of individual cases on parameter estimates. All Cook's distance values fell below a conservative cutoff of 4/(*N*–*k*–1), or 0.17 in the current sample (Fox, 2016). Visual inspection of an influence plot confirmed no cases had both high leverage and large residuals. Thus, all cases were retained in the final model.

### 3.3 Multiple regression analysis

Summary statistics for the multiple regression analysis are presented in Table 2. Maternal supportive responses significantly predicted toddlers’ regulation of PA during the gift delay task. There was a moderate positive association (β = .38, *p* = .01), such that infants who received a greater proportion of supportive responses were better able to delay touching the gift in toddlerhood. This association remained (β = .37, *p* = .03) after controlling for toddler age and family income. Neither covariate significantly predicted toddlers’ delay times.

**TABLE 2:** Multiple regression analysis predicting toddlers’ regulation of PA (delay times).

Predictor	*b*	*se*	95% CI	β	*p*
Step one	*F*(1, 25) = 4.29, *p* = .049, *R* ^2^ = .15				
Supportive responses	.79	.28	[.21, 1.36]	.38	.010
Step two	*F*(3, 23) = 2.03, *p* = .138, *R* ^2^ = .21, Δ*R* ^2^ = .06				
Supportive responses	.77	.33	[.09, 1.45]	.37	.029
Toddler age	17.83	14.38	[–11.91, 47.57]	.23	.227
Family income	1.12	4.75	[–8.70, 10.94]	.05	.815

## 4 DISCUSSION

The present study investigated whether maternal responses to infant PA at 11–12 months predict toddlers’ regulation of PA at 18–20 months. Consistent with our hypothesis, infants who received a greater proportion of supportive maternal responses showed better regulation of PA in toddlerhood, as measured by latency to touch a desirable gift. This prospective association remained after accounting for toddler age and family income. The findings provide preliminary evidence that supportive responses to infant PA are associated with emerging capacities to regulate PA.

Although previous studies have established prospective associations between parents’ emotion socialization behaviors and children's regulation of *negative* affect (Eisenberg et al., 1999; Hong et al., 2021; Li et al., 2019; Perry et al., 2020; Spinrad et al., 2004; Zinsser et al., 2021), far less is known about parenting behaviors that predict children's regulation of PA. The present findings align with theoretical frameworks proposing that supportive responses to PA—such as matching, amplifying, or elaborating on a child's joy—encourage children to share and savor positive emotions in ways that are personally or socially adaptive and, over time, may facilitate effective regulation of PA in a variety of settings (Diamond & Aspinwall, 2003; Doan et al., 2023). Empirically, our findings converge with Fredrick et al. (2019), who found that mothers’ supportive responses to adolescents’ PA during a positive event planning task were concurrently associated with adolescents’ effective regulation of PA. The present study extends this association back to infancy and demonstrates a temporal sequence in which supportive maternal responses precede later PA regulation.

Our findings are also consistent with the broader literature on observed parenting behaviors and toddlers’ delay of gratification. Maternal sensitivity (Conway, 2020; Razza & Raymond, 2013), limit setting (Houck & Lecuyer‐Maus, 2004), and distraction support (Senehi & Brophy‐Herb, 2021) have each been linked with longer delay times in early childhood.

Several observational studies with school‐age children have reported null (Moran et al., 2019; Yap et al., 2008) or unexpected (Shin et al., 2023) associations between parental responses to PA and children's emotion regulation. Methodological differences may help explain these discrepancies. Prior observational studies have often relied on brief, structured laboratory tasks (e.g., discussing a positive event for 5 min), which may constrain the range of behaviors observed and reduce ecological validity. In contrast, we observed maternal responding during a 30‐min naturalistic home observation spanning multiple contexts (divided attention, toy play, and no‐toy play). Observing mothers over a longer period and across varied contexts may have provided a more representative sample of how they typically respond to infant PA. Additionally, the coding system presented here used micro‐level coding to identify multiple types of supportive and nonsupportive responses, and all response types were combined to create a composite measure of supportive responding. This approach may have increased its predictive validity relative to coding systems focused on narrow response categories (e.g., punishing and dysphoric responses), linguistic content alone, or macro‐level/global ratings.

### 4.1 Strengths, limitations, and future directions

In addition to methodological strengths noted above, this study makes substantive contributions to the literature on parental emotion socialization. First, it addresses a notable gap by focusing on children's regulation of PA as an outcome, a dimension of emotion regulation that has been underrepresented in developmental research, despite its established relevance for children's socioemotional functioning (Doan et al., 2023). Second, the longitudinal design from infancy to toddlerhood allowed us to test prospective associations across a key developmental window, when self‐regulatory skills undergo rapid growth and are especially malleable to parenting influences (Chronis‐Tuscano et al., 2022; Doan et al., 2023; Feldman, 2003). By extending previous findings to an earlier developmental period, our findings may have implications for early identification of risk and effective prevention of PA dysregulation.

At the same time, this study has several limitations that warrant discussion and provide directions for future research. The sample size was small, limiting statistical power and confidence in the findings. Given the lack of extensive research on maternal socialization of infant PA, these findings should be considered preliminary. The small sample size also precluded examination of theoretically relevant moderators, such as infant temperament, parental attachment, and broader cultural factors like norms regarding the expression of PA (Eisenberg, 2020; Eisenberg et al., 1998). In terms of generalizability, it is noteworthy that the present sample was relatively low risk and demographically homogeneous, with most mothers identifying as White, university educated, and middle class. Although this limits generalizability of findings to families experiencing greater adversity, examining emotion socialization within low‐risk samples may elucidate how normative differences in parenting contribute to typical variation in child development (Atkinson, 2012). The demographic homogeneity also limits generalizability of findings to culturally diverse populations, where parental responses to PA and their impact on child outcomes may differ (Kiel & Kalomiris, 2015; Raval et al., 2019). Future research should evaluate whether associations between parental responses to PA and children's regulation of PA hold across varying levels of family risk and diverse cultural contexts.

Measurement considerations also warrant attention. The gift delay task assesses only one aspect of PA regulation (i.e., the ability to inhibit approach toward a desirable object) and therefore does not capture toddlers’ full repertoire of regulation abilities. Future studies would benefit from multi‐method assessments of PA regulation, such as a combination of behavioral paradigms and caregiver reports (Spinrad et al., 2007). Relatedly, the gift delay task is not a pure measure of PA regulation. Delayed gratification tasks can elicit frustration or disappointment alongside PA, and successful performance may involve the coordinated regulation of positive and negative emotions (Diamond & Aspinwall, 2003). As such, it remains unclear whether supportive responses to infant PA uniquely predict toddlers’ regulation of PA, or whether they predict self‐regulation more broadly. Future research should compare performance on delayed gratification tasks with other measures of emotion regulation to determine the extent to which task performance reflects PA regulation specifically verses broader regulation.

A final limitation is that the current study did not assess maternal responses to infant negative affect. Therefore, it is impossible to conclude whether supportive responses to infant PA represent an emotion‐specific socialization strategy or a broader pattern of supportive responding that extends across emotional contexts. Few studies have examined parental responses to both positive and negative affect within the same sample. Assessing both forms of emotion socialization together would help clarify their unique and joint contributions to toddlers’ emerging self‐regulation.

### 4.2 Implications and conclusion

From a clinical perspective, the current findings suggest that efforts to strengthen children's emotion regulation may benefit from considering how parents respond to infant PA. This is consistent with parenting interventions that emphasize sensitive responding to positive *and* negative child cues (e.g., Coyne et al., 2019; Lojkasek et al., 2008). Theoretically, findings underscore the importance of examining the regulation of PA as a distinct developmental process. Such an emphasis aligns with frameworks calling for a more balanced understanding of how both positive and negative emotions are socialized and regulated across development (Davis & Suveg, 2014; Diamond & Aspinwall, 2003; Doan et al., 2023).

In sum, this pilot study provides preliminary evidence that supportive maternal responses to infant PA are associated with better regulation of PA in toddlerhood. By examining an understudied aspect of emotion regulation, extending prior findings back to infancy, and using ecologically valid observational methods, the findings advance our understanding of how early parenting behaviors may shape children's capacity to regulate high‐arousal positive emotions. Continued research in this area has the potential to inform prevention and intervention efforts aimed at fostering emotional development from the earliest stages of life.

## CONFLICT OF INTEREST STATEMENT

The authors declare no conflicts of interest.

## Data Availability

Measures and code are available from the corresponding authors (G.S. or L.A.) upon request. Participant data are not publicly available. Participants consented to the sharing of individual (de‐identified) data with research collaborators only. We are ethically and legally obligated to honor this agreement.
